# Laparoscopic One Anastomosis Gastric Bypass as a Revisional Procedure After Failed Vertical Banded Gastroplasty: Our Center Experience

**DOI:** 10.1155/jobe/4161005

**Published:** 2025-03-30

**Authors:** Hosam Elghadban, Ashraf Shoma, Emad Abdallah, Ahmed Negm, Elsayed Abdullah, Hossam Hamed, Sameh Ghareeb, Ahmed Lotfy, Ahmed Taki-Eldin

**Affiliations:** ^1^General Surgery Department, Faculty of Medicine, Mansoura University, Mansoura, Dakahlia, Egypt; ^2^Anesthesia Department, Faculty of Medicine, Mansoura University, Mansoura, Dakahlia, Egypt; ^3^General Surgery Department, Faculty of Medicine, Horus University-Egypt, New Damietta, Egypt

**Keywords:** bariatric surgery, laparoscopic, obesity, one anastomosis gastric bypass, revisional surgery, vertical banded gastroplasty

## Abstract

**Background:** Vertical banded gastroplasty (VBG) was historically a popular restrictive bariatric procedure, but long-term failure rates due to weight regain, stenosis, and gastroesophageal reflux have necessitated revisional interventions. One anastomosis gastric bypass (OAGB), also known as mini-gastric bypass, has emerged as a viable revisional option due to its technical simplicity, lower complication rates, and promising metabolic outcomes. This study evaluates the safety, efficacy, and outcomes of OAGB as a revisional procedure following failed VBG, based on our center's experience and a review of the current literature.

**Methods:** Seventy-one patients who underwent revisional OAGB after failed open VBG between February 2014 and February 2020 were included in this retrospective study. Three years outcomes regarding weight loss (the percentage of excess body weight loss (EBWL %) and change in body mass index (BMI)), co-morbidities resolution, morbidity, and mortality were assessed.

**Results:** The EBWL % after revisional OAGB was 68.2 ± 9.4%, 65.9 ± 2.5%, and 59.6 ± 7.4% after 1, 2, and 3 years, respectively. The mean BMI before revisional surgery was 41.8 ± 3.7 kg/m^2^,which decreased to 31.9 ± 4.2 kg/m^2^ 3 years after the revisional surgery. After 1 year, there was a remarkable resolution of obesity-related co-morbidities, the remission of type 2 diabetes mellitus was 85.7%, and of hypertension was 80%. Remission of other comorbidities was also observed. Bile reflux was encountered in 6 cases (8.5%), two of them required surgical intervention.

**Conclusions:** OAGB is a feasible and effective revisional procedure after failed open VBG. However, the risk of bile reflux should be considered to justify these findings; further prospective randomized controlled trials are required.

## 1. Introduction

In recent years, the prevalence of obesity has increased significantly. Obesity is associated with multiple health risks, such as type 2 diabetes mellitus (T2DM), hypertension (HTN), osteoarthritis, obstructive sleep apnea syndrome (OSAS), and premature death, and there is no efficient nonsurgical treatment, especially for cases of morbid obesity. Several surgical procedures have been developed and modified, such as gastric bypass, gastroplasties, laparoscopic adjustable gastric banding (LAGB), and laparoscopic sleeve gastrectomy (LSG) [1–3].

Even though the gastric bypass proved to be a safe and efficient weight loss procedure, several surgical societies chose to use more conservative methods involving different types of gastroplasties. Mason in 1982 described open vertical banded gastroplasty (VBG). It was a widely utilized technique that has been the most popular in Europe for many years because the procedure leads to alimentary restriction and loss of weight while avoiding the bypass of any part of the digestive tract and its potential malabsorptive effects [4, 5].

However, VBG is associated with many long-term complications. Such as regurgitation, vomiting, and intolerance to solid food due to gastric pouch outlet stricture or a narrow band. These complications will force the patients to change their diet to semi-liquid and liquid food with high calories, which finally leads to weight regain. Another long-term complication is dilatation of the gastric pouch, which results in increased food intake and weight regain [1, 6].

Long-term follow-up after VBG demonstrated that more than 50% of the cases required revisional surgery [4–6] due to regain of weight and/or complications [6, 7].

However, revisional surgery proved to be moderately efficient regarding the loss of additional weight, has a higher rate of morbidity and mortality, is associated with technical difficulties when compared to the primary procedure, and also requires a more experienced surgeon [8, 9].

Roux-en-Y gastric bypass (RYGB) is the gold standard revisional surgery. Nevertheless, the loss of weight after revisional RYGB is lower when compared with the primary RYGB. Also, the rate of complications like leak, bleeding, and infection after revisional RYGB is from 11.8% to 16.6%, and the incidence of internal herniation is from 3.3% to 4.9%. The advantages of revisional RYGB outweigh its risks; however, the rate of complications is still high, and it is associated with long-term nutritional deficiencies [9–12]. Rutledge in 2001 reported the first 1274 cases of one anastomosis gastric bypass (OAGB) as an alternative to RYGB because it is technically simple, reversible, and has a shorter operative time [13].

As a primary bariatric procedure, OAGB is superior to RYGB regarding loss of weight and remission of T2DM and is associated with a shorter operative time and a lower incidence of postoperative complications [14].

Bariatric surgery remains the most effective treatment for severe obesity, but primary procedures such as VBG have shown limitations over time, requiring revisional interventions. OAGB, with its single gastrojejunal anastomosis and metabolic advantages, has gained acceptance as a revision strategy for failed restrictive procedures. This study discusses the indications, surgical techniques, perioperative outcomes, and long-term results of revising open VBG to OAGB.

## 2. Patients and Methods

A comprehensive review of patients undergoing revisional OAGB after failed open VBG was conducted at our center. Data on patient demographics, indications for revision, perioperative complications, weight loss outcomes, and resolution of comorbidities were analyzed. Additionally, a literature review of PubMed-indexed studies on this topic was performed to contextualize our findings.

Patients who underwent open VBG and met any of the following criteria were included: failure to achieve > 50% excess body weight loss (EBWL) within 2 years after surgery, regain of > 15% of the nadir weight in 1 year despite dietary counseling, having VBG-related complications like persistent vomiting due to stomal stenosis, severe gastroesophageal reflux, resistant stomal ulcer, or persistence or recurrence of obesity-related comorbidities were included. Patients with eating disorders, psychiatric illnesses, previous gastric operations apart from the primary bariatric surgery, and patients with incomplete medical records were excluded. The Institutional Review Board of Mansoura Faculty of Medicine approved the study before the collection of data.

## 3. Surgical Technique

All cases received antithrombotic measures in the form of LMWH 12 h before the surgery according to the Caprini risk score with intraoperative and postoperative below-knee elastic stocking. One dose of preoperative antibiotic was given to each case (Ceftriaxone 2 gm, IV) 1 h before the intervention. The patient was positioned in reverse Trendelenburg position with the legs being separated (French position). A five-trocar method was utilized; the first trocar was inserted under direct vision using the Endopath Xcel bladeless trocar (Ethicon Endo-Surgery Inc., Cincinnati, OH, USA) in the right lumbar region away from the midline incision. Adhesolysis using a vessel sealing device once entered the abdomen facilitates the introduction of other ports. The anterior wall of the stomach was separated from the left lobe of the liver and the overlying omentum. The angle of His was dissected till the exposure of the left crus. The lesser sac was entered at the level of the crow's foot, and the retrogastric space was carefully dissected. A gastric pouch was created by applying a single horizontal staple line just above the mesh, and we stopped at least 1 cm away from the previous VBG stapling line (not crossing) using an Endo GIA stapler with a 60-mm green Endo GIA stapler cartridge (Covidien, Plymouth, MN, USA). Then, a 36-French bougie was passed into the stomach, and a gastric pouch was constructed along the bougie by applying a serial vertical staple cartridges up to the angle of His using a 60-mm blue Endo GIA stapler cartridge (at least 10-cm pouch was used to perform OABG, while in cases with shorter pouches the RYGB was used, and these cases were not included in our study).

OAGB was performed by measuring 180 cm of the small intestine from the ligament of Treitz, and a gastrotomy and enterotomy were created by a hock at the anterior wall of the stomach and small intestine, and then a linear-stapled anastomosis was created between the gastric pouch and the small bowel using a 60-mm blue Endo GIA stapler cartridge. The stapling defect was closed with a single layer of Vicryl 2/0 continuous sutures. The afferent biliopancreatic limb was suspended to the new gastric pouch to make an anti-reflux mechanism to minimize possible bile reflux. Methylene blue examination as a leak test was routinely used. Careful hemostasis was done, a drain was placed, and finally the trocar sites were closed with Vicryl 0.

### 3.1. Postoperative Care

All patients were discharged to the *high-dependency unit* for 24 h, and they were on “nil by mouth” and oral intake was started by sips of clear fluids after regaining intestinal sounds, then low-calorie clear liquids for a week, then low-calorie semisolid meals for 2 to 4 weeks. After that, the patients went on a full diet and were advised to do regular physical exercise. Thromboprophylaxis (LMWH 40 units once daily) was continued until discharge. Most patients were discharged from the hospital on the second postoperative day after the gastrografin study to exclude the leakage. Patients received proton pump inhibitors for 12 months postoperatively to prevent marginal ulcers, and they were advised to stop smoking. Also, multivitamins and minerals were prescribed.

### 3.2. Outcome Measures

Patients' descriptive data before revisional surgery included age, sex, body mass index (BMI) before revisional surgery, indication of revisional surgery, and comorbidities.

The primary outcome was the percentage of excess body weight loss (EBWL %) at 12, 24, and 36 months of follow-up; patients' ideal body weight was calculated based on a BMI of 25. Anthropometric measurements were performed by the surgeon or specialized bariatric nurse.

The secondary outcomes were the operative time, intraoperative complications (such as bleeding, visceral injury, and conversion to open surgery), postoperative complications like bleeding, anastomotic leak, intra-abdominal abscess, wound infection, and port site hernia, gastric ulceration, anastomotic stricture, bile reflux, and nutritional deficiencies. We also evaluated the length of hospital stay and improvement or resolution of obesity-related comorbidities. Improvement is defined as normalization of clinical and laboratory data with a lower dosage of medication, while resolution is normalization of clinical and laboratory data without medications. Patients were followed up after 7 and 14 days after surgery. Then, follow-up visits were at 1, 3, 6, and 12 months, then yearly for 3 years after surgery.

### 3.3. Statistical Analysis

The results were collected and analyzed using the SPSS statistical package, version 22 (SPSS Inc., Chicago, Illinois, USA). Categorical data were expressed in frequency and percentage, while metric data were expressed as mean ± standard deviation.

## 4. Results

This retrospective study was conducted at the Department of General Surgery, Mansoura University Hospital, between February 2014 and February 2020. Seventy-five patients underwent revisional laparoscopic OAGB after failed open VBG, and four of them were excluded. The excluded cases were one patient with an eating disorder, another patient with psychiatric illnesses, and two patients with incomplete medical records. So, data analysis was performed on 71 patients.

Data from our center suggest that OAGB offers significant weight loss, improvement in obesity-related comorbidities, and manageable complication rates. The most common indications for revision included weight regain (52.1%), inadequate weight loss (26.8%), and severe gastroesophageal reflux (19.7%). Mean EBWL % at 12 months postrevision was 68.2 ± 9.4%, with notable improvement in diabetes and hypertension. Bile reflux was observed in a minority of cases, most of them requiring medical management.

The mean age was 39.3 ± 13.5 years, and 78.9% of the cases were females. The BMI before revisional surgery was 41.8 ± 3.7 kg/m^2^, the indications for revisional surgery and comorbidities are listed in (Table 1).

### 4.1. Primary Outcome

The baseline weight of the patients before revisional surgery was 115.5 ± 11.2 kg. The EBWL % at 12 months was 68.2 ± 9.4% and at 24 and 36 months was 65.9 ± 2.5% and 59.6 ± 7.4%, respectively. The BMI before revision was 41.8 ± 3.7 kg/m^2^, which decreased to 30.4 ± 5.3 kg/m^2^ at 12 months after surgery, and at 24 and 36 months was 30.8 ± 6.5 kg/m^2^ and 31.9 ± 4.2 kg/m^2^, respectively (Table 2).

### 4.2. Secondary Outcomes

The operative time was 126 ± 29.2 min. Six (8.5%) of the patients developed early postoperative complications (less than 30 days after revisional surgery); postoperative bleeding occurred in one patient and was managed conservatively by two units of blood transfusion. Two patients developed postoperative leakage, and upper GI endoscopy was performed for both of them. One of them was treated with stent insertion, and the other showed normal endoscopy, so laparoscopic exploration was performed and revealed an iatrogenic perforation in the omega loop that was closed by a primary suture. One patient developed an intra-abdominal abscess and was managed by US-guided tube drainage and antibiotics according to culture and sensitivity. Wound infection occurred in two cases that were treated conservatively by drainage, frequent daily dressing, and antibiotics according to culture and sensitivity. There was no conversion to open procedure, and no mortality was recorded. Hospital stay was 5.3 ± 2.6 days (Table 3).

Regarding late complications (more than 30 days after revisional surgery), bile reflux occurred in six patients (8.5%) who were diagnosed clinically and by endoscopic findings; four of them were controlled by medical treatment, and two required surgical intervention in the form of side-to-side enteroenterostomy. Two patients (2.8%) developed gastric ulcer and were managed by medical treatment. Anastomotic stricture was encountered in one patient (1.4%) and was managed by upper GI endoscopy and balloon dilatation. Malnutrition was observed in six cases (8.5%) and was managed by medical treatment. Three patients (4.2%) developed port-site incisional hernia, and they were managed by mesh repair. There was one case of mortality (1.4%) as one patient committed a suicide (Table 4).

After 12 months of revisional surgery, resolution of T2DM and HTN was 85.7% and 80%, respectively. Resolution of other comorbidities also occurred (Table 5).

## 5. Discussion

The current study was conducted to assess OAGB as a revisional surgery after failed open VBG regarding its safety, technical feasibility, and outcomes. VBG was one of the preferred bariatric surgeries as it leads to loss of weight without the bypass of any part of the digestive tract and consequently it avoids the malabsorptive effects. However, the long-term results of VBG regarding weight loss are not satisfactory because more than 20% of patients tend to regain weight. Weight regain could be attributed to disruption of the staple line, dilation of the pouch, and change in patients eating habits to sweet eaters [4, 15].

Revisional surgeries are technically demanding and complicated and are associated with a higher risk of postoperative complications than primary procedures, with perioperative morbidity rates ranging from 19% to 50% [16].

RYBG was the preferred revisional procedure following failed VBG because it can produce good weight loss results while also allowing for comorbidity adjustments [17]. However, revisional RYGB is a technically demanding surgery that is associated with greater morbidity and mortality rates [18].

OAGB is an effective revisional procedure for sustained weight loss in morbidly obese patients, and it results in resolution of co-morbidity in over 70% of cases. OAGB is a safer method than RYGP for revising failed primary restrictive bariatric procedures because it requires only one anastomosis, so it is technically simple, has a shorter operative time, has a shorter learning curve, lower incidence of anastomotic leaks and internal hernias [19, 20].

Laparoscopic revisional procedures require longer operative time as they are technically more challenging because of the hostile surgical field due to adhesions and fibrosis from the previous surgery. In our study, the mean operative time was 136 ± 39.2 min. This was in harmony with the results of previous studies by Salama et al. and Oliver et al. who reported operative time of 145.41 ± 29.18 min and 130.20 ± 47 min, respectively [21, 22], while Almalki et al. showed a longer operative time of 167.7 ± 55.8 min [12].

The key finding of our study was the weight loss after revisional OAGB. The EBWL % at 12 months was 68.2 ± 9.4%, and at 24 and 36 months, it was 65.9 ± 2.5% and 59.6 ± 7.4%, respectively. The BMI before revision was 41.8 ± 3.7 kg/m^2^, which decreased to 30.4 ± 5.3 kg/m^2^ at 12 months after surgery, and at 24 and 36 months, it was 30.8 ± 6.5 kg/m^2^ and 31.9 ± 4.2 kg/m^2^, respectively. Comparable results were reported in other studies. Almalki et al. mentioned EBWL % of 76.8 ± 57.1% and 73.0 ± 68.3% after 1 and 5 years of follow-up respectively, and BMI before revision of 37.8 ± 9.6 kg/m^2^, which decreased to 27.2 ± 6.2 kg/m^2^ and 27.8 ± 6.7 kg/m^2^ after 1 and 5 years of follow-up, respectively. Also, Sabry et al. stated that the BMI before revisional surgery was 42.80 ± 3.43 kg/m^2^, which decreased after 1 year of follow-up to 32.51 ± 3.31 kg/m^2^ [12, 23]. The loss of weight after revisional OAGB was better than after revisional RYGB, which could be explained by the longer biliopancreatic limb and the better restrictive effect of the long-sleeved gastric bypass [24].

One of the serious complications of OAGB is bile reflux, which is defined as bilious vomiting and/or the presence of bile in the esophagus on upper GIT endoscopy with the presence of GERD-like symptoms. [25]. Bile reflux can lead to gastric and esophageal mucosal damage, Barrett's esophagus, and gastric and esophageal cancer [26].

Mahawar et al. highlighted the risk of bile reflux following OAGB, suggesting that persistent exposure of the esophageal mucosa to bile may promote Barrett's esophagus and increase the risk of adenocarcinoma [27]. Scozzari et al. demonstrated that chronic bile exposure leads to oxidative stress, promoting intestinal metaplasia and gastric mucosal atrophy—recognized precursors of gastric cancer [28]. Also, Hong et al. provided a piece of evidence that bile acids can induce oxidative DNA damage in esophageal cells, promoting neoplastic transformations, especially in the context of chronic reflux [29].

Data in the literature suggest that the incidence of bile reflux after revisional OAGB is higher than that typically observed in primary OAGB procedures. This aligns with the findings of Ferrer-Márquez et al., where revisional surgeries, due to altered gastric anatomy, disruption of anti-reflux mechanisms, and potential changes in motility, show a greater predisposition to bile reflux [30].

Bile reflux is not symptomatic in all cases, and symptoms can be managed by medical treatment in most of the patients. In the current study, we encountered six cases (8.5%) of bile reflux; two of them required surgical intervention in the form of side-to-side enteroenterostomy. Comparable results of symptomatic bile reflux, which required revisional surgery after revisional OAGB, were reported in previous studies with surgical intervention options either conversion to RYGB or Braun enteroenterostomy [31–33]. A higher incidence of bile reflux after revisional OAGB compared to revisional RYGB was reported by Robert et al. and Poublon et al.; however, Robert et al. concluded that the higher incidence of bile reflux after revisional OAGB did not influence the quality of life or revision rate [14, 31].

The 8.5% incidence of bile reflux reported in the current study with the increased oncogenic potential of chronic bile reflux requires further investigations to evaluate the long-term safety of OAGB, especially as a revisional procedure. Regular endoscopic surveillance is recommended for early detection of bile-induced mucosal changes, particularly in symptomatic patients; moreover, in high-risk patients, alternative revisional procedures (e.g., Roux-en-Y gastric bypass) might be considered to reduce bile exposure.

Another important concern after OAGB is the nutritional adverse events; in the current study, we faced six cases (8.5%) with malnutrition, and none of them required re-intervention. The incidence of malnutrition could be attributed to the longer bypass limb in OAGB, which aggravates the malabsorptive effect, as well as the poor compliance of the patients to take the recommended vitamins and minerals supplements and the unhealthy feeding habits of the patients. Poublon et al. stated that the incidence of malnutrition after OAGB was 4.9%, while the YOMEGA trial reported a higher incidence of malnutrition after OAGB of 21%. This could be attributed to the longer BP limb length used in the YOMEGA trial 200 cm compared to 180 cm in our study [14, 31].

The achieved weight reduction after revisional OAGB was also reflected in the good control of obesity-related comorbidities. In the current study, the remission rate of T2DM was 85.7%. Our results were in accordance with the results of Bruzzi et al. and Debs et al. who reported 85% and 76.9% resolution rates of T2DM after revisional OAGB, respectively. On the other hand, Chiappetta et al. mentioned a 100% resolution of T2DM after revisional OAGB versus 60% resolution after revisional RYGB [34–36].

Revisional OAGB also improves other comorbidities, such as HTN, with remission rates ranging from 58% to 94% 5 years after the revisional surgery [33]. Our study showed 80% resolution of HTN. Debs et al. concluded that HTN was resolved in 82% of patients after 5 years of follow-up after revisional OAGB [35]. Also, in a study by Chiappetta et al., the resolution of HTN was 66.7% after 1 year of OAGB following failed LSG [36]. Similar results were mentioned by Bruzzi et al. with 58% resolution of HTN following revisional OAGB [34]. In the contrary, a lower rate (40%) of resolution of HTN after revisional OAGB was reported by Musella et al. [32].

Another beneficial effect of body weight reduction was an improvement in the lipid profile.

Previous studies showed that the rate of remission of dyslipidemia after revisional OAGB was ranging from 56% to 80.0% [31, 32, 34, 36]. Our results were in line with the results in the literature with a 70.6% remission of dyslipidemia.

Regarding the effects of revisional OAGB on OSAS, the present study showed a remission rate of 70%. Bruzzi et al. concluded that there was no significant difference regarding the resolution of OSAS after the primary or the revisional OAGB with a remission rate of 50% in both groups [34]. Also, in a study by Piazza et al., they stated a 66% resolution after revisional OAGB [20], while Debs et al. mentioned a higher rate of resolution of OSAS of 82% of patients [35].

Our study revealed a 72.7% resolution of GERD; the risk of GERD and bile reflux after revisional and primary OAGB is a matter of debate in the literature [37, 38]. A meta-analysis by Kermansaravi reported that revisional OAGB can lead to improvement of GERD in approximately 82% of cases [39]. However, there are other studies that reported de novo GERD in the patients who had no GERD symptoms before revisional OAGB [35, 40, 41], this could be attributed to a defective surgical technique with a short gastric pouch [42, 43]. Also, the presence of undetected or nonrepaired hiatal hernia could be another predisposing factor [44].

The results of the current study regarding weight loss, resolution of comorbidities, and rate of complications after revisional OAGB are in line with those stated in the literature for revisional RYGB [45–48]. Furthermore, Poublon et al. concluded that revisional OAGB is superior to revisional RYGB as a management procedure for insufficient loss of weight and weight regain after failed restrictive procedures with lower early complications and more weight loss [31]. Also, Khewater et al. reported that revisional RYGB following open VBG is time-consuming, technically challenging, and associated with a higher risk of conversion to open surgery. The above-mentioned reasons support the use of OAGB as a revisional procedure after failed open VBG [49].

The retrospective design of the study, which may lead to selection bias, the relatively short follow-up, the low follow-up data at 3 years, and the small sample size are limitations of our study.

## 6. Conclusion

Laparoscopic OAGB proves to be an effective revisional surgery after failed open VBG in terms of weight loss and metabolic outcomes. However, the non-negligible risk of bile reflux (8.5%), coupled with its potential long-term oncological risks, necessitates careful patient selection, long-term follow-up, and consideration of alternative procedures in high-risk individuals. Further prospective, randomized controlled trials with longer follow-up periods are essential to clarify the true incidence of bile reflux and its long-term consequences, including the risk of gastrointestinal malignancies.

## Figures and Tables

**Table 1:** Patients' demographics and preoperative data.

	No = 71
Age (years) (mean ± SD)	39.3 ± 13.5

*Sex [n (%)]*	
Male	15 (21.1%)
Female	56 (78.9%)

Weight before revisional surgery (kg)	115.5 ± 11.2

Height (cm)	163.8 ± 5.8

BMI before revisional surgery (kg/m^2^) (mean ± SD)	41.8 ± 3.7

*Comorbidities [n (%)]*	
Type2 DM	14 (19.7%)
HTN	21 (29.6%)
Dyslipidemia	18 (25.4%)
OSAS	10 (14.1%)
GERD	11 (15.5%)

*Indication for revisional surgery [n (%)]*	
Inadequate weight loss	19 (26.8%)
Weight regain	37 (52.1%)
Intractable GERD	14 (19.7%)
Food intolerance	1 (1.4%)

Abbreviations: BMI, body mass index; GERD, gastroesophageal reflux disease; HTN, hypertension; OSAS, obstructive sleep apnea syndrome; Type2DM, type 2 diabetes mellitus.

**Table 2:** Postoperative weight loss.

Months after revisional surgery	
12 months	No = 66
BMI (mean ± SD)	30.4 ± 5.3
EBWL % (mean ± SD)	68.2 ± 9.4

24 months	No = 53
BMI (mean ± SD)	30.8 ± 6.5
EBWL % (mean ± SD)	65.9 ± 2.5

36 months	No = 38
BMI (mean ± SD)	31.9 ± 4.2
EBWL % (mean ± SD)	59.6 ± 7.4

Abbreviation: BMI, body mass index; EBWL %, percentage of excess body weight loss.

**Table 3:** Operative and early postoperative outcomes.

	No = 71
Operative time (min) (mean ± SD)	136 ± 39.2

*Early postoperative complications*	
Bleeding (*n* [%])	1 (1.4%)
Leakage (*n* [%])	2 (2.8%)
Intra-abdominal abscess (*n* [%])	1 (1.4%)
Wound infection (*n* [%])	2 (2.8%)
Mortality (*n* [%])	0

Hospital stay (day) (mean ± SD)	5.3 ± 2.6

**Table 4:** Late complications.

	No = 71
Biliary reflux (*n* [%])	6 (8.5%)
Gastric ulcer (*n* [%])	2 (2.8%)
Anastomotic stricture (*n* [%])	1 (1.4%)
Malnutrition (*n* [%])	6 (8.5%)
Port-site incisional hernia (*n* [%])	3 (4.2%)
Mortality (*n* [%])	1 (1.4%)

**Table 5:** Resolution of comorbidities after 12 months (No = 66).

	Before revisional surgery	12 months after revisional surgery	Percentage of resolution (%)
Type2 DM (*n* [%])	14	2	85.7
HTN (*n* [%])	20	4	80
Dyslipidemia	17	5	70.6
OSAS	10	3	70
GERD	11	3	72.7

Abbreviations: GERD, gastroesophageal reflux disease; HTN, hypertension; OSAS, obstructive sleep apnea syndrome; Type2DM, type 2 diabetes mellitus.

## Data Availability

The data that support the findings of this study are available from the corresponding author upon reasonable request.
